# Event and Comment

**Published:** 1917-02

**Authors:** 


### DENTAL REGISTER

Vol. LXXI FEBRUARY, 1917 No. 2

### EVENT AND COMMENT

JOURNAL OF THE NATIONAL DENTAL ASSOCIATION. At last the long talked of national journal has materialized, as a monthly journal. While in form it differs little—except in size—from its predecessor, its attitude of permanence gives a new character and meaning to its very appearance. While many suggestions have been made as to its physical construction the sentiment which has produced the organ is the culmination of a long contention for a journal in some form that is owned and controlled absolutely by the dental profession. The more thoughtful and experienced members of the profession have not expected a revolution in professional ideals or practices when this event was consummated, and they will not be disappointed in the realization. No members of the profession will welcome this new and long sought opening for free and untrameled professional intercourse more than the editors and publishers of the so-called trade journals. And we predict none will surpass these same individuals in loyal support of this effort, so long as it is used for the upbuilding of the dental profession in its scientific and professional resources. We congratulate the trustees of the National Association on their wisdom in planning so wisely, and thank them for the sacrifices all associated with this matter have and may be called upon to make in the permanent establishment of the journal with such apparent promises of abundant success. We congratulate the new editor for the patient and most capable manner in which he has done his work, and on the great opportunity he will have to lead our professional activities in the future development of the profession's literary endeavors. We are confident he will be equal to the enormous task before him, if the members of the profession give him the support he will need and which they alone can give him. Dr. King has the elements of a leader, and best of all, he is saturated with the highest spiritual ideals of life and service, and we predict for him a useful and successful career. That he may have the wisdom and capacity for his task is our most cordial desire.

ERRATA. On page 493 of the October REGISTER, we published what we supposed was Dr. Hillyer's Catechism, in regard to the construction of bridges, with regard to stress and strain. We learn from Dr. Hillyer that he is not entitled to this credit as he only quotes Dr. H. E. S. Chayes, who is the author. We apologize for our mistake, but Hillyer is capable of such an expression.

